# Plant-based diets and depression: epidemiological evidence, biological mechanisms, and implications for prevention

**DOI:** 10.3389/fnut.2026.1763010

**Published:** 2026-02-26

**Authors:** Han-Ni Li, Yao Gao, Ze-Kun Li, Jiao-Jiao Qiao, Yi-Xuan Peng, Sha Liu, Xin Yan

**Affiliations:** 1 Drug Clinical Trial Institution, First Hospital of Shanxi Medical University, Taiyuan, China; 2 Nursing College, Shanxi Medical University, Taiyuan, China; 3 Department of Psychiatry, First Clinical Medical College/First Hospital of Shanxi Medical University, Taiyuan, China; 4 Shanxi Key Laboratory of Artificial Intelligence Assisted Diagnosis and Treatment for Mental Disorders, First Hospital of Shanxi Medical University, Taiyuan, China

**Keywords:** biological mechanisms, depression, diet quality, plant-based diet, plant-based diet index

## Abstract

Depression is a leading global mental health burden, and diet has emerged as a modifiable risk factor. This narrative review summarizes evidence about the association between plant-based dietary patterns and depression. It focuses particularly on diet quality and potential mechanisms. We examined plant-based diets—defined by the Plant-Based Diet Index (PDI), healthy PDI (hPDI), and unhealthy PDI (uPDI)—and plant-forward dietary patterns such as the Mediterranean, DASH, and MIND diets in relation to depressive symptoms or diagnosed depression. In diverse populations, greater adherence to healthful plant-based dietary patterns that emphasize minimally processed plant foods typically correlates with reduced depressive symptoms, better mental health, and improved quality of life. Conversely, diets that are high in ultra-processed, energy-dense plant foods are associated with a higher risk of depression. Proposed mechanisms include reduced systemic inflammation, beneficial modulation of the gut microbiota and the microbiota–gut–brain axis, and improved intake of key nutrients and phytochemicals involved in monoamine neurotransmission, neurotrophic signaling, and oxidative stress defense. In general, the influence of plant-based diets on depression seems to be more closely related to diet quality and nutrient adequacy rather than merely the elimination of animal foods.

## 1 Introduction

Depression represents a prevalent mental health condition marked by a sustained state of low mood, accompanied by a range of emotional, psychological, and somatic symptoms. Globally, the prevalence of depression is approximately 5% (1). In the female population, the incidence is slightly elevated in Africa and the Americas (5.8%) while exhibiting a lower rate in the Western Pacific region (4.2%). Conversely, for males, the highest prevalence is recorded in Africa (4.8%), whereas the Western Pacific region shows the lowest incidence (2.8%) (1). Onset age also varies regionally: around 26 years in high-income countries and earlier (about 24 years) in low- and middle-income countries (1). Depression is projected to become the leading cause of disease burden worldwide by 2030 (2). Depression etiology involves genetic, environmental, psychological, and physiological factors. Risk factors include alcohol dependence, childhood trauma, chronic physical illnesses (such as diabetes, cardiovascular disease, or obesity), female sex, low socioeconomic status, older age, personal or family history of depression, recent childbirth, and the occurrence of stressful life events (3). It is crucial to recognize and tackle these risk factors in order to develop more effective intervention strategies.

Currently, growing attention is being paid to dietary interventions for depressive symptoms. Dietary patterns are important modifiable environmental factors. Among them, plant-based diets are regarded as a healthy dietary approach. Some studies show that plant-based diets can reduce the risk of stroke, diabetes, heart disease, and all-cause mortality (4–6). Importantly, the health benefits of plant-based diets mainly stem from the consumption of nutritious plant-based foods rather than solely from the elimination of animal-derived products. Plant-based diets are inherently heterogeneous, ranging from patterns rich in minimally processed plant foods (e.g., whole grains, legumes, vegetables, fruits, and nuts) to patterns dominated by refined grains, added sugars, and other ultra-processed plant foods. Accordingly, when interpreting associations between plant-based diets and depressive symptoms, it is essential to account for dietary quality and to distinguish among different plant-based dietary subtypes. In this review, we define a plant-based diet as one that entirely or predominantly omits foods derived from animals, encompassing vegan and vegetarian diets (7). Distinct from these, we also discuss plant-forward dietary patterns such as Mediterranean, DASH, and MIND diets, which emphasize plant foods while including substantial animal products.

Preliminary studies suggest that plant-based diets may improve depression through multiple mechanisms. Among them, chronic inflammation is implicated in the pathogenesis of depression (8). Fruits and vegetables are abundant in bioactive compounds, such as polyphenols, vitamins with antioxidant properties, essential minerals, and dietary fiber. Their consumption has been shown to reduce inflammation and oxidative stress levels (9). Furthermore, the gut microbiota plays a significant role in the pathophysiological mechanisms underlying depression, with studies showing that specific bacterial species may influence mood and cognitive function (10). Plant-based diets can improve the composition and function of gut microbiota, producing positive effects on mental state (11). Additionally, plant-based diets may influence neurotransmitter synthesis and neurotrophic factors such as BDNF through micronutrients and phytochemicals. However, the specific mechanisms by which plant-based diets affect depression require further investigation.

At present, some studies have shown that plant-based diets reduce depressive symptoms, but the findings remain mixed. Some studies fail to establish a correlation between plant-based dietary patterns and depression. This may be due to differences in dietary quality and study populations (12, 13). Therefore, we aim to evaluate and better understand how plant-based diets influence depression, and provide reliable evidence for clinical practice.

## 2 Plant-based diet and depression

### 2.1 Indicators for assessing plant-based diet

Early research defined plant-based diets as vegetarian diets, categorizing subjects by whether they consumed animal foods. Currently, studies use dietary indices developed by Satija et al. (14) to assess plant-based diets. These include the Plant-Based Diet Index (PDI), healthy Plant-Based Diet Index (hPDI), and unhealthy Plant-Based Diet Index (uPDI). Plant-Based Diets is assessed by scoring food groups based on consumption frequency. By scoring food groups based on consumption frequency, these indices allow plant-based patterns to be evaluated not only by plant or animal composition but also by diet quality (Table 1).

**Table 1:** Summary of major studies on plant-based diet indices and depression.

Study (author, year)	Design	Population	Depression assessment	Adjustment	Main findings	Reference
Qi et al. (2023)	Cross-sectional study; CLHLS 2018	Older Chinese adults (aged ≥65 years), *n* = 11,623	CES-D-10	Demographics, socioeconomic status, health behaviors, physical status	Higher PDI associated with lower odds of depression; higher hPDI associated with lower odds; higher uPDI associated with higher odds.	(15)
Haghighatdoost et al. (2023)	Cross-sectional study	Iranian adults (aged ≥18 years), *n* = 2,033	HADS	Age, sex, energy, marital status, physical activity level, smoking	PDI and hPDI: no significant association; higher uPDI associated with higher odds of depression.	(16)
Vasmehjani et al. (2015)	Cross-sectional study	Iranian adolescent girls (aged 12–18 years), *n* = 733	BDI	Age, energy intake, BMI percentile, physical activity, menstrual status, parents’ occupations	PDI and hPDI: no significant association; higher uPDI associated with higher odds of depression.	(19)
Zhou et al. (2025)	Retrospective cohort study; CLHLS (2008–2018)	Older Chinese adults (aged ≥65 years), *n* = 1,666	Five-item questionnaire	Age, gender, ethnic group, smoking, drinking, exercise, education, etc	Three depressive symptom trajectories were identified; the highest vs. lowest PDI quintile was associated with lower odds of moderate/highly progressive trajectories, whereas the highest vs. lowest uPDI quintile was associated with higher odds of these trajectories.	(20)
Zhang et al. (2025)	Cross-sectional study	Chinese adults (aged ≥45 years), *n* = 3,153	PHQ-9	Age, sex, education, household income, marital status, physical activity, etc.	PDI: no significant association; higher hPDI associated with lower odds of depression; higher uPDI associated with higher odds	(37)
Wu et al. (2023)	Prospective cohort study; UK Biobank data	UK adults (aged 39–72 years), *n* = 180,532	Hospital inpatient records	Age, sex, BMI, smoking status, alcohol intake, education, etc.	PDI: no significant association; higher hPDI associated with lower odds of depression; higher uPDI associated with higher odds	(38)

This table summarizes studies examining associations between plant-based diet indices and depression outcomes, categorized by study design. CLHLS, Chinese Longitudinal Healthy Longevity Survey; CES-D-10, 10-item Center for Epidemiologic Studies Depression Scale; HADS, Hospital Anxiety and Depression Scale; BDI, Beck Depression Inventory; PHQ-9, 9-item Patient Health Questionnaire.

#### 2.1.1 PDI and depression

PDI reflects overall plant food intake levels and helps to identify the health effects associated with plant-based diets. Furthermore, it highlights the importance of a diverse range of plant foods. In this index, plant-based foods receive positive scores, whereas animal-based foods receive negative scores. The total PDI is calculated as the sum of scores across all food groups.

Results on the relationship between PDI and depression are inconsistent. This may be due to ignoring differences in plant-based diet quality. Some studies demonstrate a protective effect of PDI against depression, including research on Chinese elderly populations (15). However, other studies show no significant association between PDI and depression. For example, a cross-sectional study from a multicenter community trial found no significant association between PDI and depressive or anxiety symptoms (16). Additionally, a Spanish population-based survey study that defined plant-based diet adherence as complete avoidance of meat, fish, and animal products based on a food frequency questionnaire found this adherence associated with increased risks of depressive symptoms and stroke (17). However, such binary definitions may obscure differences in food quality and processing within plant-based dietary patterns. Therefore, current research shows contradictory results. One possible reason is that participants with high PDI may consume both healthy plant foods and substantial amounts of less healthy plant foods, such as refined grains and high-fat or high-oil items. Taken together, these mixed results suggest that plant-based classification alone may be insufficient, and that diet quality likely drives much of the variability in observed associations.

#### 2.1.2 hPDI and depression

The hPDI emphasizes healthy plant foods including whole grains, vegetables, and fruits, which are nutrient-dense. It highlights not only the quantity of plant foods but also their quality. The hPDI is calculated by assigning positive scores to healthy plant foods and negative scores to unhealthy plant foods and animal foods.

Most studies have found that higher hPDI is linked to lower depressive symptoms and reduced anxiety risk. This protective effect of a healthy plant-based diet on mental state is consistent across different populations. A systematic review found that adherence to a healthy plant-based diet was associated with lower anxiety, depressive symptoms, and psychological distress among adults (18). This suggests that healthy plant-based diets may influence psychological well-being by improving nutritional status. Additionally, a study of Iranian adolescent females found that a higher hPDI was associated with lower insomnia rates (19). However, a multicenter cross-sectional study found no significant association between hPDI and depression or anxiety (16). This may be related to differences in study design and scale selection. Despite some inconsistent findings, numerous studies support that dietary patterns rich in healthy plant foods have a protective effect against depression.

#### 2.1.3 uPDI and depression

The uPDI assesses intake of unhealthy components in plant-based diets. These include processed plant foods, high-sugar and high-fat plant foods. The uPDI assigns positive scores to unhealthy plant foods and negative scores to healthy plant foods and animal foods.

Although plant-based diets are generally considered healthy, not all plant-based foods are equally nutritious or beneficial. For example, some processed plant-based foods may contain high levels of sugar, salt, and saturated fat. Excessive intake of these components is closely associated with a range of health issues, such as obesity, diabetes, and cardiovascular diseases. Most studies show that a higher uPDI significantly increases depression risk. For example, a 10-year study found that people with the highest uPDI were more likely to experience moderate or highly progressive depression trajectories (20). A study of middle-aged and elderly people also found that uPDI was significantly associated with depression risk (15). Therefore, while plant-based diets may support psychological well-being, consumption of unhealthy plant foods may have adverse effects. Together with the hPDI findings, these results collectively demonstrate that food processing level and nutrient composition, rather than plant-based classification per se, determine mental health.

### 2.2 Association between plant-based diet and depression

#### 2.2.1 Plant-based diet and depression

Plant-based diets are characterized by reduced or no animal food intake. Vegan diets exclude all animal-derived foods, whereas vegetarian diets encompass several subtypes defined by the selective inclusion or exclusion of specific animal foods (21), including pescatarian and lacto-ovo vegetarian diets. Well-planned vegetarian diets benefit health by controlling weight, blood sugar, and blood lipids, bringing metabolic and cardiovascular benefits (22). However, findings on vegetarian diets and depression are mixed (Table 2). Some studies link vegetarian diets to increased depressive symptoms (23, 24), and several meta-analyses also report a higher risk of depression among vegetarians and vegans (25–27). Several factors may underlie these associations. Self-selection is plausible, as individuals with depressive symptoms may adopt vegetarian diets in an attempt to improve health. In addition, the cultural background also matters because when vegetarianism is uncommon, limited food availability, reduced social support, or minority stress may contribute to higher depressive symptoms. Motivations for adopting vegetarian diets and the degree of dietary planning also vary across populations, contributing to heterogeneity in diet quality and nutrient adequacy (28). Collectively, these factors may help explain the observed associations, which are unlikely to reflect a direct causal effect of vegetarian diets alone.

**Table 2:** Summary of key studies on plant-based diets and depression.

Study (author, year)	Population	Diet definition	Depression assessment	Main finding	Reference
Cross-sectional studies					
Saintila et al. (2024)	Peru adults; *n* = 768	Vegetarian (milk, eggs, derivatives, vegetables) vs. non-vegetarian (red meat and derivatives, poultry, fish, vegetables); Self-report questionnaire	PHQ-2	Higher depressive symptoms	(23)
Al Jassem O et al. (2024)	Lebanon adults; *n* = 483	Vegan/vegetarian vs. omnivore; self-report questionnaire	PHQ-9	Higher depressive symptoms	(24)
Meta-analyses					
Fazelian et al. (2022)	*k* = 13 studies (cross-sectional studies)	Vegetarian subtypes (vegan/lacto-ovo/pesco/semi-vegetarian) vs. non-vegetarian (study-defined)	Depression scales (e.g., CES-D, DASS, HADS, EPDS, PHQ-9)	Higher depression risk; No mean score difference	(25)
Ocklenburg et al. (2021)	*k* = 13 studies; *n* = 49,889	Vegetarian (vegan + vegetarian combined) vs. non-vegetarian	Depression scales (e.g., DASS-D, CES-D, PHQ-4, EPDS)	Higher depressive symptoms	(26)
Iguacel et al. (2021)	*k* = 13 studies (8 cross-sectional studies, 2 prospective, 2 randomized clinical trials, and 2 non randomized trials); *n* = 17,809	Vegetarian (lacto-ovo vegetarian) and/or vegan vs. omnivore (study-defined)	Depression scales (e.g., DASS-D, CES-D, HRSD)	Higher depression risk; No mean score difference	(27)
Askari et al. (2022)	*k* = 13 studies (4 cohort studies, 9 cross-sectional studies); *n* = 147,964	Vegetarian diets/patterns (vegan/lacto-ovo/pesco or PCA-derived vegetarian pattern) vs. omnivore/non-vegetarian; self-report/FFQ/PCA	Depression scales (e.g., GDS, CES-D, EPDS, HADS, M-CIDI)	No association	(29)
Mixed design					
Lavallee et al. (2019)	Cross-sectional: Germany *n* = 2007; Russia *n* = 3,020; USA *n* = 3,038. Longitudinal: Germany students *n* = 1,608; China students *n* = 12,744	Vegetarian (yes: no meat/fish; no meat but fish; vegan) vs. non-vegetarian; single-item self-report; all “yes” collapsed	DASS-21 depression subscales	No association overall; Slight increase over time in Chinese students	(28)
Prospective cohort studies					
Shen et al. (2021)	Taiwan adults; *n* = 10,577	Vegetarian (yes: no meat/fish) vs. non-vegetarian; FFQ + self-report questionnaire	NHIRD claims (ICD-9-CM 296.2/296.3/300.4/311)	Lower depressive symptoms	(30)

This table summarizes studies examining associations between plant-based diets and depression outcomes, categorized by study design. PHQ, Patient Health Questionnaire; CES-D, Center for Epidemiologic Studies Depression Scale; DASS, Depression Anxiety Stress Scales items; HADS, Hospital Anxiety and Depression Scale; EPDS, Edinburgh Postnatal Depression Scale; DASS-D, Depression Anxiety Stress Scale-Depression subscale; HRSD, Hamilton Rating Scale for Depression; GDS, Geriatric Depression Scale; M-CIDI, Munich Composite International Diagnostic Interview; NHIRD, National Health Insurance Research Database; ICD-9-CM, International Classification of Diseases, Ninth Revision, Clinical Modification; FFQ, Food Frequency Questionnaire; PCA, Principal Component Analysis; k, number of studies; n, total participants.

Nevertheless, some studies find no significant association between vegetarian diets and depression or anxiety (29), and cross-cultural differences have also been reported. For example, in some Western regions such as the United States, Russia, and Germany, vegetarian diets have not been clearly linked to mental problems, whereas in China they have been associated with higher levels of anxiety and depressive symptoms (28). Conversely, some studies also show vegetarian diets reduce depression risk. A prospective cohort study of 12,062 participants in Taiwan found that vegetarians had lower depression risk than non-vegetarians (30). Another cohort study on depressive symptoms and vegetarianism found that depression was associated with excluding any food group (21). The risk of depression was higher when excluding meat, fish, or vegetables, and increased further as more food groups were excluded, regardless of the specific types. Taken together, no solid evidence shows vegetarian diets per se directly lead to depression. Rather, vegetarians may lack certain nutrients. To improve dietary balance, diets should include key components such as iron, calcium, magnesium, potassium, vitamins (especially B12, B2, and D), quality protein, and unsaturated fatty acids (31).

#### 2.2.2 Other dietary patterns and depression

The Mediterranean, DASH, and MIND diets all emphasize high intake of plant foods such as vegetables, fruits, and whole grains. However, as these patterns also include specific animal-based foods such as fish, dairy, poultry, we consider them plant-forward rather than strictly plant-based. Studies have shown that they are all closely linked to reduced depressive symptoms (32–34). In a longitudinal study on dietary patterns and depressive symptoms, a diet characterized by an abundance of vegetables, olive oil, whole grains, fruits, and fish, alongside moderate consumption of wine, red meat, and processed meats, was consistently linked to a reduction in depressive symptoms (35). These dietary patterns commonly emphasize dietary diversity and high-quality plant foods. In contrast, Western diets high in processed foods, red meat, sugar and low minimally processed plant foods are linked to increased depressive symptoms, and this association becomes strengthens over time (36). Therefore, these findings likely reflect the health effects of plant-forward diet quality.

In summary, associations between plant-based dietary patterns and depression vary and appear to depend on context, motivations for dietary choice, and the degree of dietary planning. Across dietary patterns, maintaining dietary diversity and nutrient adequacy is a key consideration when interpreting depression-related outcomes.

### 2.3 Epidemiological evidence: plant-based diets and depression

#### 2.3.1 Research on older populations

Depression in older adults is a major public health challenge in aging societies worldwide. It significantly increases disease burden, functional impairment, and difficulty managing chronic diseases, while raising suicide risk. Early screening and intervention are urgently needed. In middle-aged and elderly populations, many studies have found significant associations between plant-based diets and depression incidence. A cross-sectional study of 3,153 Chinese adults aged ≥45 found that hPDI was associated with lower depressive symptoms, while uPDI correlated with higher risk (37). Here, the hPDI emphasizes higher intakes of healthy plant foods such as whole grains, fruits, vegetables, nuts, legumes, and vegetable oils, whereas the uPDI reflects higher consumption of unhealthy plant foods, such as fruit juices, sugar-sweetened beverages, refined grains, potatoes, and sweets or desserts. Prospective cohort evidence from the UK Biobank study further strengthens this finding, demonstrating that high hPDI was associated with reduced risks of dementia and depression, whereas high uPDI increased risk for both diseases (38). Additionally, a study found that greater adherence to hPDI was associated with better health-related quality of life in older adults, while greater adherence to uPDI was associated with poorer quality of life (39). Similarly, a cohort study from the Health and Retirement Study of 4,262 participants with a median follow-up of 7.8 years demonstrated that higher adherence to hPDI was inversely associated with multimorbidity risk (40). In another cohort study of 1,880 participants with a mean follow-up of 3.3 years, higher uPDI was associated with increased frailty risk (41). Therefore, these findings highlight the importance of plant-based diet quality.

The protective effect of healthy plant-based diets on mental state may partly come from improving overall health status. However, a study in Sardinia, Italy (42) found that diets overly focused on plant foods may increase depression risk in the elderly. This is especially true when animal foods are lacking. Depression incidence increased significantly without animal foods. Conversely, moderate animal food consumption was associated with better emotional status. This suggests that dietary patterns combining high-quality plant foods with nutrient-dense animal products may optimize psychological well-being in elderly populations.

Previous studies suggest older adults consume more fresh fruits, vegetables, plant oils, and legumes daily. Adding eggs to a healthy plant-based diet may also reduce depression risk (43). Future research should identify optimal food combinations for middle-aged and elderly populations. Intervention designs should include nutritional monitoring and individualized adjustments to help diet play its role in psychological well-being.

#### 2.3.2 Research on other populations

Plant-based diets’ effects on depression may vary in different populations. These include pregnant women, chronic disease patients, and youth population. Physiological, hormonal, and life stress differences may amplify or weaken dietary effects. Systematic evaluation of high-risk populations helps identify crucial nutritional factors, develop targeted interventions, and reduce depression risk.

##### 2.3.2.1 Pregnant women

Maternal psychological well-being is crucial for both mother and infant. However, research exploring its connection to plant-based diets remains limited, requiring further study. Prospective cohort evidence from the Chinese Pregnant Women Cohort Study of 4,139 participants demonstrated that higher adherence to a plant-based dietary pattern-characterized by greater intake of roughage, tubers, soybean milk, and bean products-was associated with a 15–16% reduction in antenatal depression risk compared to lowest adherence (44). This suggests healthy diets may protect against prenatal depression. Additionally, the study noted that other patterns, such as diets rich in animal protein and vitamins, also reduced prenatal depression risk. However, the inverse association was strongest for plant-based diets. Therefore, pregnant women should increase plant food intake in their daily diets.

Pregnancy is a period of high physiological and psychological sensitivity for women. Hormonal fluctuations, decreased sleep quality, and social role changes significantly increase depression risk. Pregnancy has high nutritional demands, but a healthy plant-based diet does not need to exclude all animal foods. A healthy plant-based diet focuses on high-quality plant foods as the core and meets protein, iron, calcium, vitamin B12, and other key nutrient needs through diverse food combinations. Therefore, increasing plant food proportion while reasonably combining animal foods or necessary supplements may effectively improve maternal mental state and reduce depression risk.

##### 2.3.2.2 Chronic disease patients

Chronic disease patients often develop depression due to disease burden and complications. Many studies have examined how plant-based dietary patterns reduce negative emotions in these patients. Research shows PDI and hPDI correlate with lower depressive symptoms in individuals diagnosed with cardiovascular disease, while uPDI correlates with higher symptoms (45). Additionally, greater adherence to hPDI may support mental health partly through better sleep quality. This benefit may arise because higher consumption of plant foods such as fruits and vegetables could be linked to sleep improvements via pathways involving melatonin and serotonin (45). Meanwhile, among women with type 2 diabetes, greater adherence to uPDI was associated with a higher risk of depression (46). Moreover, a cross-sectional study in migraine patients also found that adherence to plant-based dietary patterns (PDI) was linked to a reduced likelihood of experiencing anxiety, depression, and stress (47).

Therefore, dietary planning for chronic disease patients should prioritize high-quality plant-based foods. This approach may improve physical health while reducing psychological burden, thereby promoting overall well-being. This underscores the need to integrate dietary intervention as an indispensable component of chronic disease management.

##### 2.3.2.3 Youth population

Adolescents and college students are also high-risk groups for mental problems, especially regarding academic pressure and social adaptation. Studies show (48, 49) that adherence to plant-based dietary patterns is linked to fewer depressive symptoms in college students. Research conducted on diet and depressive symptoms among middle and high school students in Japan revealed that the regular intake of green and yellow vegetables is associated with a decrease in depressive symptoms among adolescents (50). These results underscore the significance of early dietary intervention for mental state in this population. It is recommended to increase plant foods in the diet to provide essential nutrients and help them enhance overall psychological resilience.

In summary, although studies have shown an association between plant-based dietary patterns and depressive symptoms across different populations, the epidemiological evidence base is still dominated by observational research, especially cross-sectional analyses. Therefore, these findings cannot establish a causal relationship between plant-based diets and depressive symptoms. Furthermore, patients with depression often experience changes in appetite and reduced motivation, which may in turn alter subsequent dietary choices. Third, even after multivariable adjustment, residual confounding may persist—particularly from factors such as socioeconomic status, health consciousness, physical activity, and comorbidities—which jointly influence both diet quality and mental health. Future research should prioritize prospective cohort studies and conduct interventional trials to better clarify directionality and strengthen causal inference.

### 2.4 Potential biological mechanisms linking plant-based diet and depression

**Figure 1:**
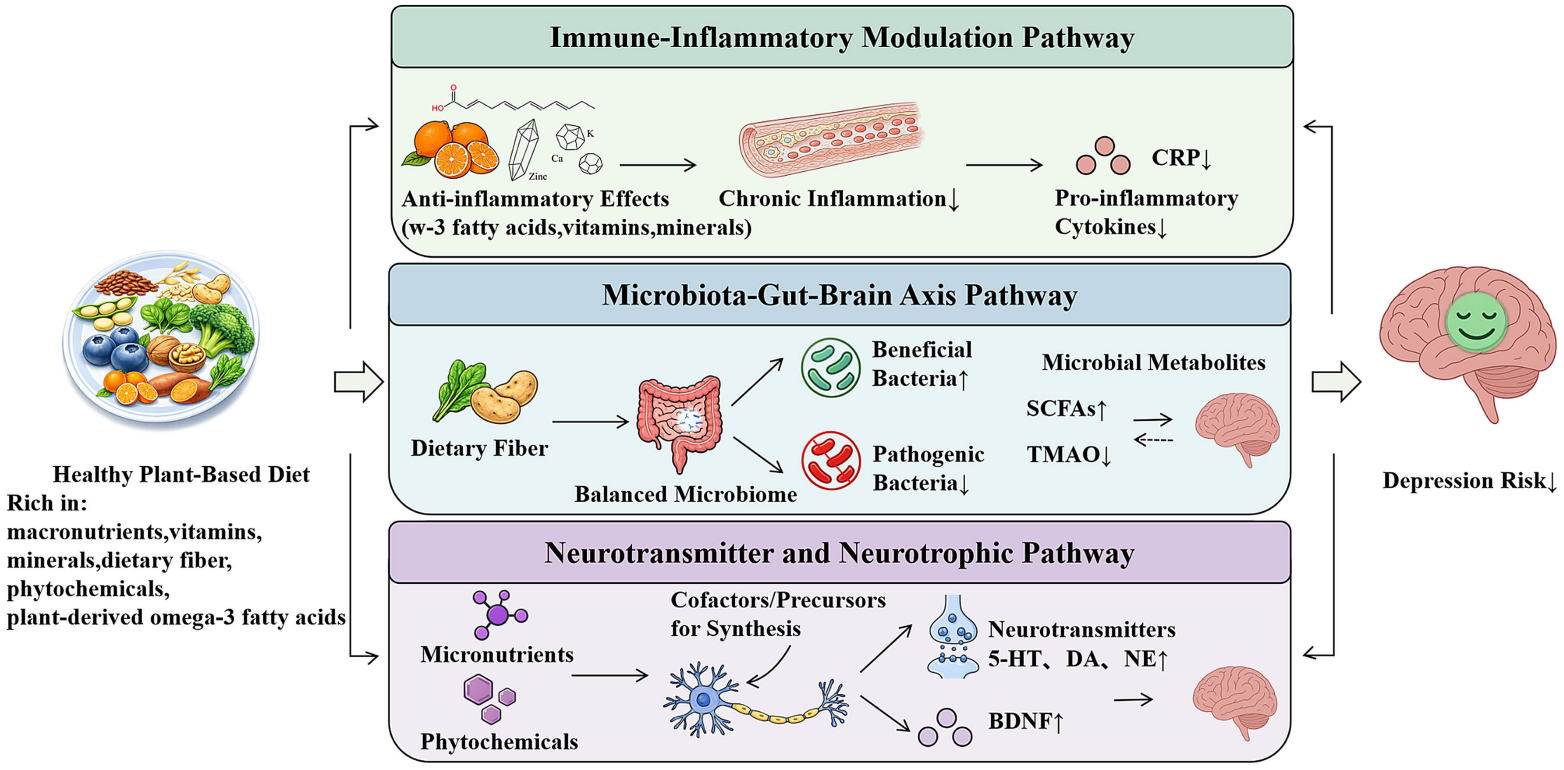
Schematic diagram: potential biological pathways linking plant-based diets to depression. Healthy plant-based diets rich in minimally processed plant foods and phytochemicals may (1) reduce systemic inflammation by lowering pro-inflammatory cytokines and inflammatory markers such as C-reactive protein (CRP); (2) modulate gut microbiota composition and function, increase beneficial bacteria, reduce pathogenic bacteria, promote SCFAs production, while reducing harmful metabolites like TMAO; and (3) support monoamine neurotransmitter synthesis and neurotrophic signaling (e.g., BDNF) by increasing intake of crucial nutrients and phytochemicals. These pathways may collectively reduce depression risk and improve mental health. All pathways depicted are hypothesized mechanisms based on current literature and require further empirical validation.

Plant-based dietary patterns have the potential to offer protective effects against depression via various biological mechanisms. Proposed mechanisms include anti-inflammatory effects, modulation of gut microbiota and the functionality of the gut-brain axis, as well as the promotion of neurotransmitter synthesis (Figure 1). Plant-based diets may offer a useful framework for hypothesis generation regarding depression prevention and adjunctive management, but these biological mechanisms require confirmation in well-designed human longitudinal and intervention studies.

#### 2.4.1 Immune-inflammatory modulation pathway

Inflammation is a protective immunological reaction that encompasses immune cells, blood vessels, and various molecular mediators. Normally, the immune system produces both pro-inflammatory and anti-inflammatory mediators. However, if the anti-inflammatory mediators cannot control pro-inflammatory responses, chronic inflammation may develop. Psychological stress can trigger inflammation responses, raising cytokine levels and affecting mood. Many studies show inflammation plays a crucial role in depression pathophysiology. Depressed patients typically have elevated pro-inflammatory cytokines including CRP, IL-6, TNF-*α*, and IL-1ra (51). Anti-inflammatory treatment can reduce depressive symptoms (52). Many plant foods contain anti-inflammatory components. Fruits, vegetables, nuts, herbs, spices, and legumes are rich sources of anti-inflammatory components, including plant-derived omega-3 fatty acids, polyphenols, vitamins, essential minerals, and probiotics (9). These may have protective effects against depression. A meta-analysis of 16 articles on high fruit/vegetable intake and plant foods’ effect on CRP showed significant CRP reduction (53). Additionally, Anthocyanin and plant oil intake also significantly reduced CRP. Plant-based diets may reduce inflammation through antioxidants and anti-inflammatory components, helping to alleviate depressive symptoms. Nevertheless, these research are based on human observational studies, and plant-based foods have indirect biomarker associations with depression. Diet-induced reductions in inflammatory markers translate to improvements in depressive symptoms are currently sparse.

#### 2.4.2 Microbiota-gut-brain axis pathway

Dysbiosis of the gut microbiota is considered a potential trigger for depression. Compared with healthy individuals, depressed patients show altered microbial diversity and shifts in the relative abundance of key bacterial groups. Firmicutes, Actinobacteria, and Bacteroidetes exhibit the most significant alterations. The increase in the Bacteroidetes/Firmicutes ratio is reflected by the enrichment of Bacteroides and the reduction of Blautia, Faecalibacterium, and Coprococcus (54). In addition, alterations in gut microbiome composition also affect microbial metabolomics. These include short-chain fatty acids (SCFAs), vitamins, and neuromodulators, which can signal through neural, endocrine, and immune pathways within the bidirectional brain-gut-microbiota axis. Notably, growing evidence shows the brain-gut-microbiota axis plays an important role in depression pathophysiology. It affects satiety, digestion, behavior, and mood. Research indicates that depressive phenotypes accompany gut microbiome changes. These changes further affect depression-like behavior and increase depression risk (55).

Diet is an important factor determining gut microbiota composition and function. In particular, plant-based diets are rich in macronutrients (dietary fiber, protein, fatty acids) and micronutrients (minerals, vitamins) that shape microbial diversity and community structure. A systematic review (56) on plant-based diets and gut microbiota found that individuals on such diets have higher Lachnospiraceae and lower Bacteroidaceae. In vegans and vegetarians, analyses of plasma microbial metabolites, including SCFAs and trimethylamine N-oxide (TMAO), further suggest that gut microbiota serves as a critical mediator in diet-host interactions. SCFAs are vital for neuronal function, nerve cell maturation, and blood–brain barrier stability (57), whereas elevated TMAO levels are linked to inflammation and metabolic diseases. Plant-based diets, rich in fiber and healthy fats, promote SCFA production and reduce TMAO levels (58). Another cross-sectional study (59) on protein intake in healthy women found that high plant protein intake was negatively associated with mental disorders, kynurenine levels, and the Firmicutes/Bacteroidetes ratio. Another study (60) found that restoring gut microbiota and gut-brain axis function with probiotics, prebiotics, and healthy diets may relieve depressive symptoms. As an adjunct to antidepressants, this approach can improve psychiatric status.

In general, plant-based dietary patterns may help regulate gut microbiota composition and function. These changes may include increased abundance or diversity of certain taxa and altered production of microbial metabolites such as SCFAs. However, direct links to depression outcomes in humans have not yet been fully characterized.

#### 2.4.3 Neurotransmitter and neurotrophic pathway

The “monoamine hypothesis” of depression suggests that imbalances in monoamine neurotransmitters such as serotonin (5-HT), dopamine (DA), and norepinephrine (NE) are crucial mechanisms. Most antidepressants restore emotional balance by modulating these monoamine systems. Some specific nutrients act as cofactors or precursors for key enzymes involved in neurotransmitter biosynthesis and related metabolic pathways. For example, folate and B vitamins are considered vital nutrients for promoting neurotransmitter synthesis. Folate participates in one-carbon metabolism—a process that donates methyl groups essential for neurotransmitter synthesis. B vitamins act as coenzymes or coenzyme precursors in amino acid metabolism, thereby affecting monoamine neurotransmitter production (61). In addition, research shows 12 antidepressant nutrients are linked to depression prevention and treatment. These include folate, iron, long-chain omega-3 fatty acids (EPA and DHA), magnesium, potassium, selenium, thiamine, vitamin A, vitamin B6, vitamin B12, vitamin C, and zinc (62). Plant foods rich in these nutrients include leafy greens, lettuce, peppers, and cruciferous vegetables, as well as animal foods include bivalves like oysters and mussels, various seafood, and organ meats (62). This supports the possibility that improving overall diet quality and micronutrient adequacy may complement established treatments.

Additionally, it should be noted that strict vegetarian diets can also cause deficiencies in nutrients essential for brain function. This may increase the risk of depression. Vitamin B12 is found almost exclusively in animal products and is crucial for DNA and neurotransmitter synthesis (63). One study found that community-dwelling elderly with Vitamin B12 deficiency or low levels at baseline had a higher likelihood of developing new-onset depression 4 years later (64). Moreover, iron serves as a cofactor for key enzymes in dopamine and serotonin synthesis. Plant-based foods contain mainly non-heme iron, which has lower absorption efficiency than heme iron. Iron deficiency may cause low mood and fatigue. In a cross-sectional study of 1,875 participants aged 65 and older, researchers found that depressive symptoms were associated with anemia and low serum ferritin levels (65). Equally important, Omega-3 fatty acids, particularly long-chain EPA and DHA, are primarily derived from fish oil (66). They are important components of neuronal cell membranes and have anti-inflammatory effects while regulating immune system and neural signal transmission (67, 68). Plant-derived Omega-3, mainly alpha-linolenic acid (ALA), converts to EPA and DHA in the body with low efficiency. This may lead to insufficient intake in vegetarians (69). Some evidence suggests omega-3 supplementation may be beneficial as an adjunctive therapy for depression in some people (70). Therefore, the impact of plant-based diets on depression largely depends on whether they can ensure adequate supply of these key nutrients while avoiding animal products.

Besides neurotransmitters, brain-derived neurotrophic factor (BDNF) is vital for neuronal survival, growth, and synaptic plasticity. BDNF levels are usually low in patients with depression (71). A meta-analysis found that polyphenol supplements significantly raised BDNF levels in healthy young and middle-aged adults (72), and this effect was linked to better cognitive function. Plant foods contain many phytochemicals, including polyphenols, flavonoids, and carotenoids, that show potential for preventing and treating depression. Studies show polyphenols can relieve depressive symptoms, but more research is needed (73).

In summary, while micronutrient adequacy and phytochemical intake from plant foods may theoretically support neurotransmitter metabolism and neurotrophic signaling, direct evidence that these pathways mediate the relationship between plant-based dietary patterns and depression outcomes in humans is largely absent. Prospective intervention studies are needed to validate these mechanisms.

### 2.5 Future research directions

Growing evidence suggests plant-based diets may benefit mental state, particularly in depression prevention and intervention. Future research should include additional rigorous prospective cohort studies and randomized controlled trials. These studies should clarify how different plant-based diets and their specific components independently contribute to depression risk. This will enhance our comprehension of the underlying mechanisms of action. Additionally, the relationship between plant-based diets and biomarkers such as inflammatory markers, gut microbiota, and neurotransmitter metabolism requires further investigation. The causal relationships of these biological mechanisms remain unclear. More research is needed to explore the specific effects of plant-based diets on these biomarkers and their mechanisms in depression. This will provide a more solid scientific basis for future application of plant-based diets in depression intervention.

## 3 Conclusion

Based on this review, current research primarily derived from observational studies with varying designs and regional populations does not demonstrate a unidirectional causal relationship between plant-based diets and depression. The effects depend on multiple factors, including diet quality, individual health status, lifestyle, and sociocultural background. Overall, high-quality plant-based diets are associated with lower depression risk, better mood, and higher quality of life, while unhealthy plant-based diets may increase depressive symptoms. This suggests diet quality is crucial to mental health. For potential mechanisms, several biological pathways have been proposed. High-quality plant-based diets may influence depression through reduced inflammation, improved gut microbiota diversity, and enhanced neurotransmitter synthesis. However, these proposed mechanisms remain speculative and should be interpreted as hypothesis-generating, pending further experimental validation to establish causality. Additionally, although current research supports the potential value of plant-based diets in depression prevention and treatment, studies exhibit heterogeneity in diet definitions, measurement methods, and sample composition. Future research should more systematically and precisely assess the role of plant-based diets in depression prevention and treatment.
